# Forming the Perception of WIC Infant Feeding Recommendations: A Qualitative Study

**DOI:** 10.3390/nu15030527

**Published:** 2023-01-19

**Authors:** Emily Fisher, Kathryn Wouk, Priyanka Patel, Chuanyi Tang, Qi Zhang

**Affiliations:** 1 School of Community and Environmental Health, College of Health Sciences, Old Dominion University, Norfolk, VA 23529, USA; 2 Pacific Institute for Research and Evaluation, Chapel Hill, NC 27514, USA; 3 Department of Marketing, Strome College of Business, Old Dominion University, Norfolk, VA 23529, USA

**Keywords:** WIC, breastfeeding, formula, maternal and child health, food assistance program

## Abstract

Nearly half of newborns in the United States are enrolled in the Special Supplemental Nutrition Program for Women, Infants, and Children (WIC). Promoting breastfeeding is a programmatic priority, although formula vouchers are provided for those who do not exclusively breastfeed. Previous literature suggests that participant perception of WIC’s breastfeeding recommendations is a significant factor predicting breastfeeding initiation, duration, and exclusivity outcomes. However, little is known about how participants’ perceptions of WIC’s breastfeeding recommendations are formed. To address this knowledge gap, we conducted a qualitative pilot study in Nevada, interviewing 10 postpartum WIC mothers and 12 WIC staff who had interacted with participants regarding infant feeding. Results showed participants and staff reported various perceptions of what WIC recommends, the factors that contribute to these perceptions, and how these perceptions affect breastfeeding practices. Respondents also described that WIC has a negative legacy as the “free formula program,” and that environmental factors, such as the recent formula recall, have had an impact on participants’ infant feeding practices. More effective public campaigns and programmatic strategies are needed to target participants’ prenatal self-efficacy and to communicate the availability of skilled lactation support in the early postpartum period to improve participants’ perceptions of WIC’s position on breastfeeding.

## 1. Introduction

The Special Supplemental Nutrition Program for Women, Infants, and Children (WIC) serves nearly half of all infants born in the USA [1]. Since breastfeeding is critical to both maternal and infant health outcomes [2,3,4], breastfeeding promotion and support are priorities of the program. However, WIC participants consistently have lower breastfeeding rates than non-participants, even when compared to WIC-eligible non-participants [5,6].

Prior studies have examined factors influencing WIC participants’ breastfeeding practices [7,8,9,10]. For example, breastfeeding attitudes and beliefs related to self-efficacy, intrinsic and extrinsic motivation, and social or cultural norms have been found to predict prenatal breastfeeding intentions and later breastfeeding practices [7,8,9,10]. Studies also show that many WIC participants have similarly positive attitudes toward breastfeeding and formula feeding [11] and that, compared to non-participants, WIC participants perceive formula adoption as more personally and socially acceptable due to socioeconomic needs and anticipation of breastfeeding challenges [12]. Among African American mothers, infant feeding decisions are influenced by participation in prenatal breastfeeding classes, the perspectives of people in their social network, and by historical experiences of racism contributing to breastfeeding stigma [10,12,13]. Finally, information about the cost of supplemental formula (in addition to the WIC-provided formula) is perceived as an additional motivation to breastfeed among WIC participants [14].

Beyond the above factors, Zhang et al. (2021) found that if pregnant participants perceived that WIC recommends breastfeeding and formula equally or formula only, they had significantly worse breastfeeding outcomes than those who perceived WIC as recommending only breastfeeding [15]. Furthermore, pregnant participants who perceived that WIC recommended breastfeeding only were significantly less likely to stop exclusive breastfeeding through five months or to stop any breastfeeding through 11 months [16]. Although WIC promotes breastfeeding, the availability of free formula through this program may encourage participants to perceive that WIC recommends formula feeding. This perception may be reinforced by aggressive marketing from the breast milk substitutes (formula) industry [17]. However, little is known about how participants form their perceptions of WIC’s infant feeding recommendations and what factors may affect these perceptions. 

To address the above research gaps, this study aimed to understand how WIC participants form their perceptions about the program’s breastfeeding recommendations and to identify factors that influence these perceptions. In the WIC setting, many factors, including human factors (e.g., WIC clinic staff), WIC operations (e.g., formula voucher provision), or environmental factors (e.g., the formula recall), may contribute to participants’ perceptions about the WIC program’s breastfeeding recommendations. By understanding the factors influencing WIC participants’ perceptions, the WIC program may revise or augment programmatic strategies to improve breastfeeding in WIC populations. Since little extant literature is available to address these research questions, we adopted a qualitative approach for this exploratory study. Analysis of these exploratory narratives can reveal areas for future studies to confirm and extend preliminary findings. 

## 2. Materials and Methods

To understand how WIC participants form their perceptions of WIC breastfeeding recommendations, we conducted in-depth interviews with both WIC participants and WIC staff. WIC participants were all postpartum mothers who provided retrospective data regarding their prenatal perception of WIC breastfeeding recommendations and infant feeding decision-making behaviors. The WIC employees who were interviewed all directly interacted with pregnant and postpartum WIC participants regarding infant feeding plans and practices. The interviews were recorded, transcribed, and analyzed. The Old Dominion University Institutional Review Board approved this study.

### 2.1. Subject Recruitment and Interviews

To recruit WIC participants, we collaborated with the Nevada WIC state agency. We posted a banner on the app used by participants to check their WIC benefits and assist with redemptions. Interested participants who clicked on the banner were then redirected to a Qualtrics site to complete the informed consent and screening survey. Only first-time mothers who had given birth within the last six months were eligible for this study. Researchers then contacted eligible participants, who agreed to participate in an in-depth telephone interview. 

To recruit WIC staff, an invitation was sent by email to all local WIC agencies in Nevada. Interested staff were asked to complete a Qualtrics screening survey. Researchers contacted the staff who agreed to be interviewed and conducted in-depth interviews with them via phone. 

For both types of interviews, a semi-structured guide was developed based on a literature review and the substantive expertise of research team members. Three major questions were discussed in the interviews: (1) what WIC participants think WIC recommends for infant feeding practices; (2) what factors influence participants’ perceptions of WIC’s infant feeding recommendations; and (3) what WIC participants’ actual infant feeding practices are across the postpartum period. All interviews began with broad questions and then moved to more specific questions. Interviewers probed and asked additional questions to obtain more insights into WIC participants’ perceptions and behaviors. All interviews were recorded and transcribed. No additional subjects were added after the collected data reached saturation.

### 2.2. Qualitative Analyses

The transcribed interview data were coded and analyzed following the procedures suggested by Strauss and Corbin (1990) [18]. NVivo 12 was used to assist with coding and analysis. Interviews were reviewed by the research team and an initial codebook was developed to guide inductive coding. Both staff and participant interviews were coded independently by two researchers. Any disagreement between the two coders was settled through discussion in research team meetings. To ensure the accuracy and reliability of the results, the final coding was refined through several iterative team discussions until a consensus was reached.

## 3. Results

Demographics of the WIC participant interviewees are presented in Table 1. Half of the participants were 18–24 years old. Most mothers identified as Hispanic (60%), with the remainder identifying as non-Hispanic Black (40%). They were single (60%) or unmarried but living with a partner (40%). Ninety percent of the sample had at least a high school degree or General Equivalency Diploma (GED). Only 30% of participants had a full-time job at the time of the interview. Almost all participants (90%) were at 3–6 months postpartum at the time of the interview. Among these participants, three reported exclusively breastfeeding in the hospital, five reported exclusively formula feeding in the hospital, and two reported mixed feeding practices (breastfeeding and formula feeding) in the hospital. A total of six mothers reported that they attempted to breastfeed at some point, either pre- or post-discharge from the hospital. 

The demographics of WIC staff interviewees are presented in Table 2. Like the WIC participants, two-thirds of the staff identified themselves as Hispanic. The staff all reported being at least high school graduates or having a GED. Over 80% of the staff worked full-time and had at least one year of WIC employment experience. Their positions included supervisors/managers (25%), breastfeeding coordinators working substantively on breastfeeding education and support (25%), or breastfeeding counselors working directly with participants to provide breastfeeding education and support (50%). 

We organized our findings with emerging themes from the interviews according to the five broad research questions from our interview guide. Because we interviewed both WIC participants and WIC staff, we discussed the findings generated from the participant interviews first, followed by the insights from the staff interviews. We compare major differences and similarities in the findings between participants and staff in the discussion section. 

### 3.1. What Does WIC Recommend for Infant Feeding?

#### 3.1.1. Participant Perspectives

Most WIC mothers stated they believed that WIC recommends breastfeeding. Those who perceived that WIC recommends formula indicated that they had either experienced this recommendation firsthand or had heard about it from family and/or friends. One participant reported feeling uncertain of WIC’s infant feeding recommendations but believed that WIC supported each mother’s infant feeding decision, regardless of whether it was breastfeeding or formula feeding. 

“Oh yeah, they always recommended breastfeeding.”-P17.

“My mom was just telling me about it because we were having a conversation about formula and then she was letting me know that there was this company ‘WIC’ that could help me.”-P65.

“I just think, you know, they go based off your decision; they don’t question you about it. Or they don’t mind.”-P23.

#### 3.1.2. Staff Perspectives

The staff had broader views about how participants perceive WIC’s infant feeding recommendations, including perceptions that WIC recommends (1) formula only, (2) breastfeeding only, (3) both formula and breastfeeding equally, (4) the mother’s own infant feeding choice, or (5) WIC as a source of neutral infant feeding education. Roughly half of the WIC staff speculated that participants believe WIC recommends formula for infant feeding. Three WIC staff said mothers consider WIC a safe place to get formula if they cannot exclusively breastfeed. Less than half of the WIC staff members reported that participants perceived WIC as recommending only breastfeeding and providing resources to support breastfeeding. Only a few WIC staff thought that participants perceived that WIC recommends both formula feeding and breastfeeding equally and supports mothers’ individual choices. Additionally, one WIC staff member reported that participants perceived WIC’s role as providing neutral education to help them decide on their infant feeding plan. 

“Moms associate WIC with formula because we give most of our clients formula.”-S13.

“They feel very supported, and they feel they have everything, every resource that they may need regarding breastfeeding.”-S19.

“I think that they feel that WIC tries to recommend more breastfeeding. But they also know that we can help them with formula. So, it’s kind of more like a 50–50.”-S8.

### 3.2. What Factors Influence Participants’ Perceptions of WIC’s Infant Feeding Recommendations?

#### 3.2.1. Participant Perspectives

Many factors influence participants’ perceptions of WIC’s infant feeding recommendations, but we ultimately categorized these factors into relationship and media influences. 

Among relationship influences, the most frequently reported factors were close interpersonal relationships with family members or maternal figures, comprising the participants’ mothers, cousins, and friends. A few WIC participants indicated that their family members specifically suggested they seek infant feeding assistance from WIC. They also stated that observing friends’ infant feeding behaviors was a strong influence on how they perceived WIC’s infant feeding recommendations. Organizational relationships, including those with WIC staff and healthcare providers, also played a role in influencing mothers’ perceptions of WIC’s recommendations; however, these relationships were reported less frequently. 

“There is actually one friend that I have, and she also has WIC, and she told me about how they teach you all this stuff about the healthier choices for the baby and for even me as a mom. Yeah, I have a lot of people around me that use WIC.”-P17.

“Well, my cousin told me ‘I had WIC with my two kids’, and she thinks it’s an amazing program because, you know, they’re able to provide milk for her baby. She thinks it’s a great program.” -P59.

“The staff. They… When I did my interview for WIC, it was more like getting to know one another. And she told me ‘How are you feeling?’ and the importance of breastmilk. And she even told me stories, like, about her‚—I think it was her daughter who was having difficulties, and what she did to achieve more breastmilk, and just keep on doing it as much as you can, and you’re doing a great thing for the baby.”-P86.

Media influences also played a role in influencing mothers’ perceptions of WIC recommendations. WIC mothers reported that encounters with the WIC application for mobile phones (WIC App), the WIC website, and WIC posters hanging in physical office locations all influenced their perceptions of WIC’s infant feeding recommendations. Additionally, several mothers reported that receiving a WIC breastfeeding pamphlet helped shape their views on WIC’s infant feeding recommendations. 

“I believe they had it in, like, a little pamphlet that they gave me. Other than that, I don’t know.”-P35.

“Yeah, I got, like, a little paper package in, like, that tells you, like, the milestones and, like, little charts. I believe I had got [...] that says, like, you know, like, kind of, like, baby’s feeding schedule and stuff like that.”-P45.

“In their office, like, it says, breastfeeding. They really try to showcase that breastfeeding is very healthy. Like, posters and things like that.”-P45.

#### 3.2.2. Staff Perspectives

WIC staff reported various factors that shape mothers’ views of WIC’s infant feeding recommendations, noting that interpersonal relationships, healthcare providers, and organizational associations had the strongest effects on mothers’ perceptions.

Most WIC staff reported that interpersonal relationships, consisting of family, friends, and peers, have the strongest influence on how WIC participants form their beliefs about WIC’s infant feeding recommendations. WIC staff observed that mothers value advice from family members or maternal figures and female friends regarding WIC’s services and public image. Additionally, a few WIC staff members mentioned that participants were influenced by their partners when concluding WIC’s infant feeding recommendations.

“The friends who go to WIC, who don’t breastfeed, get their formula and tell them. So, their minds just think like, ‘Oh, WIC is promoting formula because they give us formula. They give us up to nine cans of formula.’ And that’s a lot. And that’s what I think really makes people think, like, WIC is promoting formula.”-S13.

“Peers, friends, neighbors suddenly feel like if you don’t get the formula, you’re missing out on the program, you’re not getting all the benefits from WIC.”-S14.

Roughly half of WIC staff suggested that healthcare providers, such as doctors and nurses, impacted how mothers view WIC’s infant feeding recommendations. WIC staff described healthcare providers as the first to assist mothers with breastfeeding in the hospital. However, when mothers encountered early breastfeeding issues, such as concerns about milk supply, healthcare providers often suggested using formula. They consistently referred to WIC as a source of free formula. 

“Pediatricians out here for us push formula for the moms, any little issue they tell them, like, Oh, here’s a prescription for your WIC office to get formula, because the baby has a milk allergy and milk is hard to get out of your system so you can’t breastfeed.”-S13.

A small number of WIC staff recognized that WIC had a long history of being perceived as a “free formula program,” and this legacy could have a lasting impact on mothers’ perceptions of WIC’s infant feeding recommendations, even if the program has significantly improved its focus on promoting and supporting breastfeeding. 

“I think that one probably comes from a historical basis where WIC was always known as the place to get formula. And so, then it got the reputation of being the formula people. And then, but as time has gone on and WIC has really worked at revamping our image, I guess I think that might be the stem of the breastfeeding only, because the passion that comes through, and we’re talking about breastfeeding moms may receive it as we’re, like, we only want you to breastfeed, and we don’t want you to do anything else.”-S9.

### 3.3. What Factors Influence WIC Participants’ Infant Feeding Practices? 

Three infant feeding categories were explored for this study: exclusive breastfeeding, mixed feeding, and formula feeding. Exclusive breastfeeding is defined as feeding only breastmilk, either by breast or by bottle. Formula feeding is defined as feeding only formula. Mixed feeding is defined as feeding a combination of any amount of breastmilk and formula. It should also be noted that in our interviews, mothers used the word “milk” interchangeably for breast milk and formula.

#### 3.3.1. Participant Perspectives

Most mothers indicated that they had chosen to breastfeed as their intended infant feeding practice before the birth of their child. In the hospital, however, mothers reported experiencing various challenges that changed their original infant feeding plans. Several mothers indicated that their infants struggled to latch on to their breasts appropriately, either due to breast shape or an infant tongue or lip tie. Mothers also reported that difficulty with early milk production impeded their plans to breastfeed and contributed to the introduction of formula. Furthermore, a COVID-19 infection during the birthing process disrupted one mother’s plan to breastfeed by leading to the early introduction of formula.

“I tried to breastfeed, but as I said, she wasn’t latching on, and she wasn’t taking it, so she ended up being formula fed.”-P74.

“I tried, because of the C-section my milk didn’t, breastmilk didn’t come in as it should.”-P86.

“I wasn’t necessarily allowed to be in the NICU with them because they were both in the NICU, and I was COVID-positive. I was put in isolation, and I couldn’t breastfeed them. I tried pumping, and then, unfortunately, nothing was able to really come out, and what I did produce was very, very little.”-P35.

After hospital discharge, supplementing with formula or exclusively feeding formula occurred for various reasons. At least half of the mothers reported that they continued to have trouble with their infants latching on to their breasts. Mothers reported feeling that their infants were not satisfied with breastfeeding alone. Several mothers cited returning to work as the primary reason for introducing formula, specifically referencing convenience as a motivating factor. One mother felt that her breastmilk made her child’s bowel movements softer, which led her to increase formula feeding to alleviate this symptom. At the time of this study, only two mothers were using mixed feeding practices, while the others had all switched to exclusive formula feeding. 

“It was just more convenient. I had to go back to work.”-P45.

“Well, since I started work, I haven’t been able to have her latch. It’s been a struggle. They do have these pumps. I pump at work, like, twice a day, which helps because that way it doesn’t lower my supply. But that’s just how it’s affected it.”-P86.

#### 3.3.2. Staff Perspectives 

WIC staff expressed different opinions regarding whether most participants changed their infant feeding plans postpartum or whether they typically stuck to their prenatal infant feeding plan. WIC staff speculated that factors such as social support, institutional support, medical complications, body image, and workplace characteristics led some WIC mothers to adopt formula feeding even if they had intended to breastfeed. Most WIC staff thought family, peers, and friends often encouraged mothers to introduce formula as an infant feeding practice. For example, the mothers’ friends were often a factor in leading mothers to perceive WIC as endorsing formula feeding. Roughly half of WIC staff reported that cultural experiences such as family traditions and home environments changed the mother’s perception of recommended infant feeding practices. 

“Home environment and their culture and what their mothers and grandmothers did all determine the reason a mother chooses to breastfeed or formula feed.”-S5.

“One of those moms who grew up in a household or a family where no one ever breastfed, but you want to breastfeed, you might not have a realistic view of what breastfeeding looks like because your experience is only with formula-fed babies who behave much more differently.”-S9.

“It would be word of mouth, talking to a friend. ‘Hey, have you been on this program? They offer formula’ or ‘I’m on this program. They’ll help you with breastfeeding.’ I think a lot has to do with word of mouth.”-S3.

Regarding institutional support, half of the WIC staff reported that participants developed an intention to breastfeed after communicating with WIC staff during pregnancy. Furthermore, half of the WIC staff shared that they believed mothers would follow guidance from healthcare providers, such as pediatricians or nurses, to introduce formula feeding due to a perception that it would be an easier infant feeding practice than breastfeeding.

“I’ve had moms tell me, like, that because of my conversations with them that they are now willing to even try the fact of breastfeeding. I had moms tell me that I changed their perspective when it comes to formula feeding and how they think it’s best to give baby breast milk.”-S13.

“Pediatricians tell them, just bottle feed or it’s better to just bottle feed versus them trying to breastfeed because I feel like breastfeeding could be a little bit more demanding.”-S10.

Medical complications were rarely mentioned as a reason mothers introduced formula despite intending to breastfeed. A small number of WIC staff mentioned that mothers were frequently frustrated with their early postpartum milk supply due to a lack of knowledge. They observed that mothers would start breastfeeding their infants for the first few weeks and then supplement with formula due to a perception that breastmilk alone would not satisfy their infants’ needs. One WIC staff member recalled encountering a mother-infant dyad who had experienced latch issues, which led to formula introduction. WIC staff speculated that a lack of knowledge regarding appropriate latching techniques and support at home contributed to using the formula. 

Some WIC staff noted that mothers might choose formula feeding due to stigma or issues related to body image. One WIC staff member reported that returning to the workplace can lead some mothers to introduce formula to accommodate the lack of structural support or workplace resources for breastfeeding.

“A lot of times breasts are sexualized, so they don’t think, like, it’s right to put their baby at their breasts.”-S13.

“Unfortunately, because we work here in Vegas, there’s a mom that said they must go back to work immediately. And because of the type of job they do, like, let’s say, the casinos, they feel that they don’t have the structure or the ability to continue to provide for their baby, the breasts. So, it’s easier for them to go towards the formula.”-S3.

### 3.4. How Did the Formula Recall Influence WIC Participants’ Infant Feeding Practices? 

In February 2022, the Food and Drug Administration (FDA) issued a powdered infant formula recall after several consumer complaints claimed bacteria in the product caused infant illness and may have even contributed to the deaths of several infants [19]. Some WIC state agencies distributed the formula brands included in this recall. One consequence of this recall was a formula shortage in the United States for several months, which coincided with the period of data collection for this study. Therefore, we asked the participants and staff how this environmental change affected WIC participants’ views about infant feeding and their infant feeding behaviors.

#### 3.4.1. Participant Perspectives 

Several mothers recounted emotional responses to news of their infant’s formula being recalled, saying they felt scared, stressed, and frustrated. The formula recall led half of the mothers interviewed in our study to switch formula brands if they were currently using a recalled product. A few mothers inquired about re-lactation or the possibility of increasing breastfeeding practices. Only one mother, who was exclusively formula-feeding the infant and never intended to breastfeed, reported that her infant feeding practices were unaffected by the recall.

“Oh my God, it was stressful. Some of the cans I had, I think out of the thirteen cans, eight of them were part of the recall.”-P71.

“I still give the formula a chance. I have heard great things about Enfamil, so maybe I try that, but I give it a try, so just to see how it would work.”-P2.

“Not formula feed so much. And because of—I’m assuming because of that, you know, the recall, that’s why there’s been, like, Similac. But, you know, I mean, no formula. So, you have no choice at this point but to breastfeed, you know.”-P9.

#### 3.4.2. Staff Perspectives 

WIC staff members described participants’ emotional reactions to the formula recall, noting that mothers were upset, concerned, and scared for their infants’ wellbeing. Most WIC staff indicated that they had experienced an increase in mothers interested in breastfeeding and re-lactation. Three WIC staff members stated that most mothers impacted by the recall contemplated switching formula brands. 

“We’ve seen some moms that were partially breastfeeding go back to just exclusively breastfeeding. That way, they wouldn’t have to be having that concern in regards to the formula.”-S15.

“Similac formula, because we’ve already gotten some feedback from moms that they don’t want to go back to Similac after the—once the inventory is replenished. That they have no interest in going back to Similac”-S9.

“I think so, I do think more people are thinking a little more about breastfeeding as they look forward. I’ve had some moms say that, and it definitely—they had to switch up the formula they were using, which was something that was working well. A mom did not intend to switch, but when you can’t get it and baby has to eat. Many women had to switch to a different brand and things.”-S20.

### 3.5. Policy Recommendations

#### 3.5.1. Participant Perspectives

Most WIC mothers reported feeling satisfied with WIC services and offered few suggestions for improving the program. When questioned on whether they would change their infant feeding practices if WIC stopped offering free formula, most WIC mothers said withdrawing this resource would not impact their current practices. 

“No, I think I’ll continue to use formula.”-P23.

#### 3.5.2. Staff Perspectives

A third of WIC staff stated that they believed WIC succeeded in supporting mothers with their infant feeding practices and promoting breastfeeding. When the interviewers probed further regarding suggestions to better align WIC’s narrative of breastfeeding promotion with participant behaviors, most staff reported that the WIC program needed to implement more strategies targeting participant perception of WIC’s recommendations. 

“I think we’re doing a pretty good job on being open with both. Because we still see moms that feel and tell us, ‘You guys are so good, like, you don’t make me feel I’m doing something wrong if I choose formula.”-S10.

“Perception. I believe the change in perception is already in motion. I don’t know what we can do about the perception that we only push breastfeeding, because that doesn’t make any sense to me.”-S9.

The staff proposed various methods to influence participant perceptions of WIC as recommending breastfeeding, including strategies focused on internal practices and organizational relationships. They suggested that internal practices for improving perception could involve using social media platforms more effectively, meeting with participants more frequently, marketing WIC as a breastfeeding support agency, and increasing education on the benefits of breastfeeding. 

“Maybe more contacts with mom, more education. If we had more actual appointments with mom ideally in person.”-S20.

“Think we need some major PR (public relation) reform, you know, like advertising in a lot of different ways, you know, doing print advertisements, doing social media, just really getting that information out there, like, because the idea of what WIC is so different than what it was in the seventies.”-S16.

Organizational partnerships were also encouraged by the staff to improve participant perception of WIC’s breastfeeding recommendations. Staff reported that online workshops for pediatricians and other medical professionals might help them to align their breastfeeding recommendations and work together to support WIC participants to breastfeed. A third of the WIC staff believe that improved outreach and education can increase postpartum mothers’ enrollment in WIC and breastfeeding practices. Partnering with other governmental agencies like the Centers for Disease Control and Prevention was also mentioned as a strategy to improve breastfeeding rates. 

“Talking to the doctors to help us work as a team so we can change the client’s perspective or point of view. Because if the doctor tells them something else, they go with what the doctor says versus what we recommend or, be on the same page of what we both recommend.”-S10.

“Doctors should get involved and partner with WIC, or WIC should reach out and partner. I mean, we do this on the agency level, but nationally, I think when people see messaging and when, nationally, if we reached out to doctors, if we reached out to pediatricians’ offices. Maybe if we did online workshops with doctors, we could let patients know that WIC is on the same page as their doctor, and then everybody has their best interest. I think their perception of WIC—I think they’ll be more trusted.”-S7.

## 4. Discussion

This exploratory study provides preliminary evidence of how WIC participants formed their perceptions of WIC’s infant feeding recommendations and identifies potential factors that may influence these perceptions. More importantly, the findings from interviews with both WIC participants and WIC staff appear to cross-validate and complement each other in ways that can inform future research and policymaking. 

While breastfeeding is a programmatic priority for WIC, the program is also the predominant source of formula for low-income infants born in the USA, which may contribute to conflicting perceptions of WIC’s recommendations on infant feeding [20,21,22]. Most WIC participants perceived that WIC recommends breastfeeding as a normative infant feeding practice. They reported developing this perception via interpersonal relationships, mostly with maternal figures, and from media influences such as the WIC App, website, posters, and breastfeeding pamphlets. In contrast, most staff described WIC participants as perceiving that WIC recommends formula. WIC staff described mothers as perceiving WIC as a safe place to access general infant feeding information, such as guidance on using formula or resources for breastfeeding. 

Many study participants who had planned to breastfeed instead introduced formula in the early postpartum period due to problems with milk production or poor latch. These mothers did not turn to WIC for skilled lactation support to address these issues and continue breastfeeding, but rather perceived that WIC was an environment where the introduction of formula would be supported and enabled via formula vouchers. This aligns with earlier literature that has found that WIC participants anticipated barriers to breastfeeding related to milk production and latch and perceived these barriers as challenging to overcome and accepted as inevitable [12]. Formula-feeding WIC mothers have also been shown to perceive WIC primarily as a formula provider, with the remaining benefits being perceived as less valuable [23]. WIC should invest in additional strategies to communicate the availability of skilled lactation support and improve access to these services to address early breastfeeding challenges.

Similarly, during the formula recall, most WIC participants perceived WIC as a place to turn for guidance on selecting a different formula brand and not as a resource for re-lactation or additional breastfeeding guidance. Most mothers recounted experiences with switching formula brands in response to the recall, with only a small number reporting an interest in re-lactation. WIC staff and mothers reported emotional responses of concern and frustration regarding the formula recall. WIC staff reported that switching formula brands and inquiring about re-lactation and breastfeeding had increased among mothers. Given that WIC participants are more likely to use formula than eligible or non-eligible non-participants, they are also more likely to be subject to the emotional stress caused by formula shortages [17]. 

While mothers were far more likely to recount the physical problems with low milk supply and latch as major barriers to breastfeeding, WIC staff were more likely to report that the mothers’ interpersonal and medical relationships with family members, maternal figures, nurses, and doctors were the main reasons mothers introduced formula to respond to these challenges. These conflicting views between the participants and the staff may result from the fact that both participants and healthcare providers perceive that WIC endorses breastfeeding but does not provide a critical source of early lactation support when mothers face common early breastfeeding challenges. Since healthcare provider support is a well-established predictor of breastfeeding success [24], WIC should invest more time and resources in communicating WIC’s role in breastfeeding promotion and support to healthcare providers to align messaging and services. For mothers from racial and ethnic minority groups, racism has also been documented through experiences of being discriminated against or stereotyped during postpartum hospitalization, contributing to racialized disparities in who receives lactation support and/or human donor milk versus formula supplementation [25,26,27]. The WIC program should work collaboratively with major medical organizations to improve equal access to evidence-based maternity practices, such as those advanced by the Baby-Friendly Hospital Initiative, both in and outside the hospital setting [28,29].

Both WIC mothers and staff described the significance of interpersonal relationships, especially with WIC participants’ mothers and friends, in the formation of participants’ perceptions of WIC’s infant feeding recommendations. The role of social support, particularly from maternal figures and peers, has been previously identified as a critical factor in maternal decision-making regarding infant feeding [30,31,32,33]. To have a more substantial impact on infant feeding behaviors, WIC should extend breastfeeding education and promotion activities to a broader set of stakeholders in the mother’s family and community. Additionally, mothers and staff both cited a perception of the improved convenience and ease of formula feeding versus breastfeeding, aligning with earlier literature on formula-feeding mothers reporting bottle feeding as more manageable and more compatible with returning to work [9,34]. The WIC program could target this perception through increased prenatal education that improves participants’ breastfeeding self-efficacy [35,36] and collaboration with employers to improve workplace support for breastfeeding.

This study also identified future research topics that should be investigated. Prior studies [15,16] found that WIC participants’ prenatal perceptions of breastfeeding recommendations were associated with WIC participants’ breastfeeding exclusivity and duration. This study was the first to identify how the perception may be formed and the potential factors that may influence the perception. However, this study did not examine the mechanism of how these perceptions may affect breastfeeding outcomes and durations directly. One possible channel suggested by Zhang et al. (2021) is that participants’ perceptions may be associated with their breastfeeding intention, which has been proven to be an essential mediator of breastfeeding outcomes [15,37,38]. When a WIC participant has a prenatal intention to breastfeed, she may perceive WIC’s breastfeeding recommendation differently from a mother who intends to formula feed. For example, a prenatal WIC participant who has decided to breastfeed her infant is more likely to perceive WIC as recommending and supporting breastfeeding. In contrast, if a mother has determined to feed formula exclusively, she may perceive that WIC supports formula feeding since it provides the free formula. However, even after controlling for participants’ prenatal breastfeeding intention, their perception of WIC’s breastfeeding recommendations has an independent effect on breastfeeding outcomes [15,16]. Thus, future research is needed to untangle the dynamic relationship between WIC participants’ perceptions of WIC’s breastfeeding recommendation and their breastfeeding attitudes, intentions, and behaviors. 

Although many WIC participants in our study believed that WIC recommends breastfeeding, they considered the availability of free formula through WIC as providing a safe backup plan. According to Shin and Milkman (2016), having a backup plan has many benefits, such as reducing uncertainty and associated psychological discomfort among WIC participants, but it also has adverse effects [39]. For example, the availability of free WIC formula may decrease WIC participants’ desire to breastfeed, reducing their likelihood of doing so. Future research is needed to examine how the availability of free WIC formula as a backup plan affects WIC participants’ breastfeeding practices. The “backup hypothesis” deserves more research, especially in the context of predatory marketing by the infant formula industry [40]. Additionally, WIC should continue incentivizing breastfeeding through the larger package of food benefits offered to exclusively breastfeeding participants. 

This study has several strengths in its methods and significance. This is the first qualitative study to examine the participants’ perception of WIC’s breastfeeding recommendations. It is important to include WIC participants’ voices in WIC policymaking to build effective breastfeeding promotion and support strategies [41,42]. The complementary views of participants and staff provide a better understanding of a complicated issue from diverse perspectives. Moreover, the study provides the first evidence of participants’ and staff’s responses to the recent formula recall, which can inform future research and programmatic planning for supporting infant feeding in the context of different emergencies.

As a pilot exploratory investigation, findings from this qualitative research must be interpreted within the context of the study design. First, like other qualitative studies, results from this study could be biased due to the non-random sampling and limited sampling methods. However, in contrast to quantitative research, which emphasizes an adequately large sample size to generalize findings to the broader population, qualitative research uses smaller samples to inform conceptual generalization, which may not be statistically representative [43,44]. The sample size of this study is in line with other qualitative studies that have investigated breastfeeding among WIC mothers [45,46,47]. Second, this study only included English-speaking participants and lacked the views of non-English-speaking participants, which may have also contributed to the small sample size. Third, this pilot study presents interview data from WIC participants in a single U.S. state. While WIC is a federal program in which findings from a small sample of agencies may be generalizable across diverse WIC settings providing similar infant feeding services, more large-sample, mixed-methods studies are needed to confirm and extend these initial findings.

## 5. Conclusions

This exploratory study found that WIC participants and WIC staff held some conflicting and overlapping perceptions of how participants formed their perception of WIC’s breastfeeding recommendations. Factors contributing to this perception formation included the influence of key stakeholders, the mother’s social network and healthcare providers, the WIC program’s current media and breastfeeding promotional influences, and the program’s long history as a distributor of free formula. The results also provided preliminary evidence of how WIC participants and staff navigated the recent formula recall, although its impact on increasing breastfeeding among previously formula-feeding mothers appeared to be minimal. 

## Figures and Tables

**Table 1:** Characteristics of WIC Mothers Interviewed (n = 10).

Variable	Categories	Proportion (%)
Age	18–24	50
	25–29	30
	30–34	10
	35–39	10
Race/Ethnicity	Hispanic	60
	Non-Hispanic Black	40
Marital Status	Not married but living with a partner	40
	Single	60
Education Level	Less than high school	10
	High school graduate or GED	40
	Some college but no degree	20
	Associate degree	10
	Bachelor’s degree or above	20
Employment *	Full-time work	30
	Part-time work	30
	Students	10
	Homemaker	20
	Other	20
Urban/Rural Residence	Urban	30
	Suburban	40
	Rural	10
	Other	20
Post-partum Months	0–2 months	10
	3–6 months	90
Number of Infants at Birth	Singleton	80
	Multiples	20
Language Spoken at Home	English	80
	Spanish	20

* Sub-categories are not mutually exclusive.

**Table 2:** Characteristics of WIC Staff Interviewed (n = 12).

Variable	Categories	Proportion (%)
Age	18–24	8.3
	25–29	16.7
	30–34	16.7
	35–39	16.7
	40≤	41.7
Gender	Male	8.3
	Female	91.7
Race/Ethnicity	Hispanic	66.7
	Non-Hispanic White	25.0
	Non-Hispanic other	8.3
Education Level	High school graduate or GED	8.3
	Some college but no degree	33.3
	Associate degree	8.3
	Bachelor’s degree or above	50.0
	Yes	83.3
Full-time Employment	No	16.7
	<1 year	16.7
WIC Working History	1–5 years	16.7
	>5 years	66.7
	Supervisor/Manager	25.0
WIC Position *	Breastfeeding coordinators	25.0
	Breastfeeding counselors	50.0
Urban/Rural Residence	Urban	66.7
	Suburban	25.0
	Rural	8.3
Language Spoken at Work *	English	100.0
	Spanish	41.7

* Sub-categories are not mutually exclusive.

## Data Availability

Due to the sensitive nature of the interview scripts, no data will be available.
